# Whole Transcriptome Profiling of Maize during Early Somatic Embryogenesis Reveals Altered Expression of Stress Factors and Embryogenesis-Related Genes

**DOI:** 10.1371/journal.pone.0111407

**Published:** 2014-10-30

**Authors:** Stella A. G. D. Salvo, Candice N. Hirsch, C. Robin Buell, Shawn M. Kaeppler, Heidi F. Kaeppler

**Affiliations:** 1 Department of Agronomy, University of Wisconsin, Madison, Wisconsin, United States of America; 2 Department of Agronomy and Plant Genetics, University of Minnesota, St. Paul, Minnesota, United States of America; 3 Department of Plant Biology, Michigan State University, East Lansing, Michigan, United States of America; 4 DOE Great Lakes Bioenergy Research Center, Michigan State University, East Lansing, Michigan, United States of America; National Key Laboratory of Crop Genetic Improvement, China

## Abstract

Embryogenic tissue culture systems are utilized in propagation and genetic engineering of crop plants, but applications are limited by genotype-dependent culture response. To date, few genes necessary for embryogenic callus formation have been identified or characterized. The goal of this research was to enhance our understanding of gene expression during maize embryogenic tissue culture initiation. In this study, we highlight the expression of candidate genes that have been previously regarded in the literature as having important roles in somatic embryogenesis. We utilized RNA based sequencing (RNA-seq) to characterize the transcriptome of immature embryo explants of the highly embryogenic and regenerable maize genotype A188 at 0, 24, 36, 48, and 72 hours after placement of explants on tissue culture initiation medium. Genes annotated as functioning in stress response, such as glutathione-S-transferases and germin-like proteins, and genes involved with hormone transport, such as PINFORMED, increased in expression over 8-fold in the study. Maize genes with high sequence similarity to genes previously described in the initiation of embryogenic cultures, such as transcription factors BABY BOOM, LEAFY COTYLEDON, and AGAMOUS, and important receptor-like kinases such as SOMATIC EMBRYOGENESIS RECEPTOR LIKE KINASES and CLAVATA, were also expressed in this time course study. By combining results from whole genome transcriptome analysis with an in depth review of key genes that play a role in the onset of embryogenesis, we propose a model of coordinated expression of somatic embryogenesis-related genes, providing an improved understanding of genomic factors involved in the early steps of embryogenic culture initiation in maize and other plant species.

## Introduction

In order to meet global food, feed, and fiber needs in the face of climate change and predicted population growth, current and future crop improvement efforts will likely include the utilization of biotechnology-based approaches [1], [2]. This includes the discovery and functional analysis of agriculturally important genes for crop research and product development. Currently, most of the crop genetic engineering systems utilize embryogenic, regenerable tissue cultures as a critical part of the transformation process [3]. Totipotent, embryogenic cultures are also desirable for efficient somatic embryo production for other agricultural biotechnology applications such as clonal propagation, production of synthetic seed [4], and the proposed utilization of somatic embryos for gamete cycling in rapid breeding [5].

At the molecular level, it is widely accepted that the induction of somatic embryogenesis involves massive cellular reprogramming and activation of various signaling cascades [6], [7]. The necessary triggers that induce somatic embryogenesis in tissue culture are tantamount with stress response [8], [9]. As the accumulation of near-damaging cellular signals trigger change, only specific genotypes are capable of efficient cellular adaptation, pluripotency, and embryogenic competence in tissue culture [10].

The majority of crop genotypes within species display low embryogenic growth response in culture. This genotype-dependent culture response decreases the efficiency and significantly limits the application of clonal propagation schemes and current transformation systems in the genetic study and improvement of crop plants [11]. In maize, the inbred line A188, which displays a high embryogenic culture response, has been utilized in investigations on the inheritance and genetic control of the genotype-dependent culture response [12]–[14] and in improving embryogenic response efficiency and regeneration ability in tissue culture [15], [16].

Despite the agronomic importance, few genes with a direct role in the induction of somatic embryogenesis in tissue culture have been identified, and their role in embryogenic culture response in maize and other crops is not understood. In *Brassica napus*, Arabidopsis, and Chinese white poplar, the transcription factor, BABY BOOM, when ectopically expressed in recalcitrant lines in tissue culture, was shown to induce somatic embryogenesis [6], [7]. LEAFY COTYLEDON and PINFORMED genes have been thoroughly studied in zygotic embryogenesis in normal seed development with some studies suggesting that these genes may also be important to somatic embryogenesis in tissue culture [7]. In addition, regulatory genes such as AGAMOUS, WUSCHEL and CLAVATA have been studied in Arabidopsis for their role in meristem formation, somatic embryo formation, and callus maintenance [6], [7], yet their role in maize tissue culture is not well understood.

To improve the understanding of genomic factors involved in early somatic embryogenesis in maize, we examined the transcriptome of the highly embryogenic maize inbred line A188 at 0 to 72 hours (h) after placement of immature embryo explant tissues onto culture initiation medium. Some of the first embryogenesis-related alterations in cell processes and cell division that are necessary for efficient embryogenic response occur during the early initiation stages. Based on our findings, we propose a coordinated expression model for somatic embryogenesis-related genes and describe an overview of global expression trends highlighting genes that are up- and down-regulated during the time course of the study. Genes related to somatic embryogenesis in other species and the relative expression of maize genes with high sequence similarity is also discussed. This research provides important information relating to the improvement of crop tissue culture and genetic engineering systems.

## Results

### Global analysis

RNA-seq reads were generated for nine immature embryo samples consisting of 25 embryos per sample of the maize inbred line A188. Embryo samples were placed on culture initiation medium from 0 to 72 hours. In total, the number of reads per sample ranged from 11 million (M) to 36 M (Table S1). Reads were aligned to the B73 maize reference genome sequence [17] and a set of representative transcript assemblies (RTAs) missing in the B73 reference genome sequence that were identified in transcriptome analyses of 503 maize inbred lines including B73 [18]. Expression values were determined using fragments per kb exon model per million mapped reads (FPKMs) using Cufflinks [19]. Biological replicates at each time point were correlated to assess data reproducibility. Pair-wise Pearson’s correlations of expression values between embryos from two different donor plants collected at the same time point ranged from 0.9643 to 0.9927 (Table S2), indicating a high degree of reproducibility. Based on this analysis, average expression values from the two replicates were used for downstream analyses. A total of 28,992 annotated B73 reference genes and 6,405 RTAs were expressed in at least one time point (FPKM>0); while 10,464 reference genes and 2,276 RTAs were not expressed in any sample (Table S3).

The highest expressed reference genes across time points included genes that function in stress response, RNA binding, DNA synthesis and chromatin structure (Table 1). For example, GRMZM2G156632 is a highly expressed gene which is annotated as wound induced protein 1 (WIP1). Another gene related to plant defense that was among the highest expressed genes was GRMZM2G051943, which encodes for chitinase A1. The RTA with the highest expression at 0 h was joint_Locus_12721 with an FPKM value of 399.89 which decreased over 8-fold to 49.13 at 72 h. The highest RTA expressed at 36 and 48 h was joint_Locus_33043 with an FPKM value of 13.07 at 0 h and FPKM values of 309.83 and 200.60 at 36 and 48 h, respectively. This RTA was annotated as encoding IN2-1, which based on sequence similarity, is a glutathione S-transferase (GST) protein. The highest expressed RTA at 72 h was joint_Locus_83 (204.13 FPKM) which did not match any known gene annotations.

**Table 1:** Highly expressed genes in immature zygotic embryo explants in tissue culture.^a^

		FPKM					
Gene	Function	0 h	24 h	36 h	48 h	72 h	Cluster Number
GRMZM2G020940	unknown	889.44	1548.51	1168.15	1203.65	1282.68	6
GRMZM2G080603	grp1 (glycine-rich protein1)	1005.03	1816.68	2345.42	2365.11	2412.29	6
GRMZM2G480954	unknown	22.07	1217.33	993.33	844.36	571.95	6
GRMZM2G153292	tua2 (alpha tubulin2)	1292.61	1065.69	1384.88	1349.02	1313.01	6
GRMZM2G080274	ARATH HON1 Group	1205.96	164.49	251.87	293.59	288.91	6
GRMZM2G337229	ole1 (oleosin1)	1129.77	1341.73	1377.95	1155.93	812.47	6
GRMZM2G051943	chitinase A1	1.01	1636.10	2150.28	1676.96	1331.93	3
GRMZM2G332838	Histone H4	1295.95	267.26	649.95	642.20	566.99	6
GRMZM2G011523	unknown	6.47	1952.69	1191.21	825.81	451.70	3
GRMZM2G057823	ald1 (aldolase1)	1421.28	802.19	931.20	729.20	695.52	6
GRMZM2G088511	unknown	998.56	928.75	1323.82	1023.23	1005.62	6
GRMZM2G084195	Histone H4	1273.38	358.28	845.45	883.28	770.60	6
GRMZM2G091715	unknown	1207.21	495.31	568.79	534.97	466.14	6
GRMZM2G303374	unknown	954.01	888.49	1058.47	1105.93	1267.46	6
GRMZM2G152466	tua4 (alpha tubulin4)	1504.67	548.22	923.32	1070.86	1147.94	6
GRMZM2G165901	rab15 (responsive to abscisic acid15)	1063.15	2870.95	2945.72	2222.61	1919.31	6
GRMZM2G072855	Histone H4	1242.98	248.54	541.83	557.07	509.32	6
AC233865.1_FG001	Histone H4	2001.74	427.13	765.30	812.44	745.99	6
GRMZM2G031545	unknown	801.56	714.73	982.54	1193.72	1366.95	6
GRMZM2G156632	WIP1 (wound induced protein1)	2.36	7158.54	2770.89	788.17	254.05	3
GRMZM2G028393	sci1 (subtilisin-chymotrypsin inhibitor homolog1)	18.32	4544.08	2155.63	1779.17	1152.94	3
GRMZM2G126900	unknown	1.15	1308.76	571.75	490.23	435.86	3

a Fragments per kilobase of exon per million fragments mapped (FPKM) at 0, 24, 36, 48, and 72 h after placement on tissue culture initiation medium and the assigned gene cluster number determined by k-means analysis.

#### Characterization of genes with 8-fold or greater expression change

**Figure 1:**
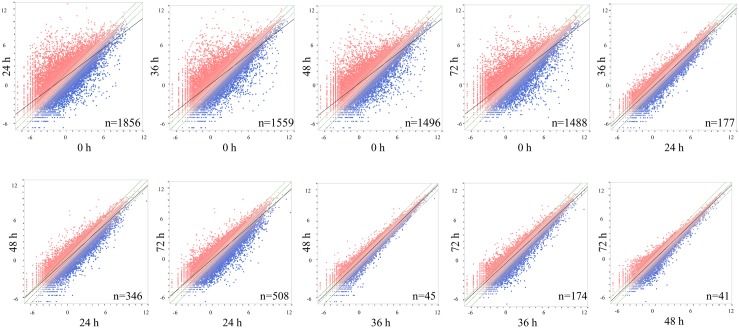
Gene expression changes in early somatic embryogenesis. Scatter plots of gene expression changes as log2 values of fragments per kilobase of exon model per million fragments mapped (FPKM) in immature zygotic embryo explants of maize inbred line A188 after placement on culture initiation medium for each time point comparison at 0, 24, 36, 48, and 72 h where n is the number of genes differentially expressed greater than 8-fold for each time point comparison. Red dots represent genes that are up-regulated, blue dots represent genes that are down-regulated, the middle green line indicates no fold change in expression, the two outer green lines indicate a 2-fold change in expression, and the solid black line is the best fit linear correlation.

In order to gain an understanding of genes expressed in this time course study, we selected genes differentially expressed by at least 8-fold compared to the control time point (0 h). Comparison of gene expression patterns across the surveyed time points indicated that the largest number of genes with a change in expression profile was from 0 to 24 h (Figure 1). This is supported by the observation that 1,856 genes were expressed at (or greater than) an 8-fold change when comparing 0 vs 24 h, 1,559 genes at an 8-fold change when comparing 0 vs 36 h, 1,496 genes at an 8-fold change when comparing 0 vs 48 h, and 1,488 genes at an 8-fold change when comparing 0 vs 72 h. Similarly, comparisons at other time points revealed 177, 45, and 41 genes differentially expressed 8-fold in comparisons of 24 vs 36, 36 vs 48, and 48 vs 72 h, respectively. Most genes differentially expressed at 8-fold were up-regulated. For example, 72%, 67%, 66%, and 72% of the genes differentially expressed when compared to 0 h were up-regulated at 24, 36, 48, and 72 h time points, respectively (Table S4). When considering a 2-fold change in expression, 8,174 genes were differentially expressed when comparing 0 vs 24 h, 6,737 genes when comparing 0 vs 36 h, 6,444 genes when comparing 0 vs 48 h, and 6,580 when comparing 0 vs 72 h.

The most abundant genes with large expression changes were enriched for biological processes such as oxidation-reduction processes, metabolic processes, protein phosphorylation, and transmembrane transport (Table S5). For example, genes with an 8-fold expression change or greater at 24 h were enriched for antiporter and transmembrane transport such as GRMZM2G006894, a hydrogen-exporting ATPase and GRMZM2G479906, GRMZM2G415529, and GRMZM2G366146 are ABC transporters. Other genes up-regulated at least 8-fold at 24 h were involved in transport of amino acids, sugars, and peptides or were specific to transmembrane transport of important nutrients. There were fewer genes that were down-regulated at 8-fold or greater. These genes were involved in membrane transport of amino acids and metals. For example, GRMZM2G140328 and GRMZM5G892495 are down-regulated 8-fold at 24 h and are both involved in calcium signaling. Genes that were up-regulated 8-fold or greater at 72 h revealed glycosyl-related genes such as GRMZM2G179063, a UDP-glucosyltransferase, and iron ion binding genes such as GRMZM2G103773, a BRASSINOSTEROID-6-OXIDASE 2. Genes down-regulated greater or equal to 8-fold between 0 h and 72 h were also enriched for genes involved in stress response such as ATP binding heat shock proteins GRMZM2G360681 and GRMZM2G310431, genes involved in nutrient assimilation such as GRMZM2G087254 and AC189750.4_FG004 both adenylyl-sulfate reductases, and genes involved in regulation of transcription such as GRMZM2G011789, a CCAAT box binding transcription factor.

#### Characterization of genes grouped by k-means analysis

**Figure 2:**
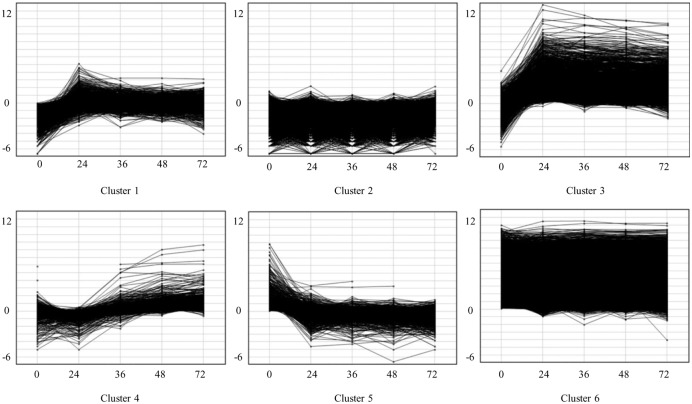
K-means clustering of genes expressed during early somatic embryogenesis. Log2 values of fragments per kilobase of exon model per million fragments mapped (FPKM) in genes with greater than zero FPKM expressed in immature zygotic embryo explants of maize inbred line A188 at 0, 24, 36, 48, and 72 h after placement on culture initiation medium grouped by expression trends as uncentered Pearson’s correlation coefficient in six k-means clusters.

The induction of somatic embryogenesis involves a complex coordination of multiple pathways [20], [21]. Genes involved in hormone response, signal transduction, stress response, transcriptional regulation and cellular reorganization have been described previously [7], [9], [20]. We sought to determine if our maize transcriptome data supported concepts and models regarding these major biological functions during the very early stages of embryogenic tissue culture initiation. Using k-means clustering with six clusters, we identified groups of genes with similar expression patterns including: (1) up-regulated and then down-regulated during the developmental window highlighted in this study, (2) both up- and down-regulated during the time course, (3) genes with an expression trend towards increased up-regulation from 0 to 24 h, (4) genes with a higher up-regulation later in the time course at 36, 48 and 72 h compared to all other genes expressed in the developmental window, (5) genes with an expression trend towards large-scale down-regulation from 0 to 24 h, and (6) genes with constitutive expression throughout the study (Figure 2 and Table S6).

Gene ontology enrichment was significant for clusters 1, 2, 3, and 6 (Table S7). Genes in cluster 1 were enriched for protein kinase and phosphorylation activity. Specifically, these genes were enriched for functions involving DUF26 signaling receptor kinases and post-translational modification receptor like kinases, as well as UDP glucosyl and glucoronyl transferases. Gene expression values in cluster 2, enriched for apopotic processes, ranged from a minimum FPKM of 0.006 to a maximum of 4.414. Since the expression of these genes in cluster 2 was very low, these FPKM values could be inaccurate and attributed to noise. Genes in cluster 3 (initially up-regulated) were involved in numerous functions including transmembrane transport activity, oxidation-reduction processes, and heme binding or iron ion binding such as cytochrome P450 related genes. Finally, genes in cluster 6 were enriched for intracellular functions such as chromatin structure and DNA synthesis, ribosomal proteins synthesis, transcription factors, cell transport and RNA processing. A total of 2,704 RTAs, the largest proportion of RTAs grouped into a k-means cluster, were grouped into cluster 6 (Table S6).

Since gene ontology enrichment was not significant for clusters 4 and 5, MapMan B73 5b gene annotations [22] were used to describe genes in these clusters. Genes in cluster 4 were related to protein degradation, signaling receptor kinases, transcription factors, and genes involved in hormone metabolism and secondary metabolism. Genes in cluster 5 were involved in similar functions to cluster 4, but in addition, some cluster 5 genes were annotated for functions in amino acid and lipid metabolism (Table S6). RTAs with annotations relating to transcription factors that promote embryo development which could be involved in somatic embryogenesis are RTA joint_Locus_9393 annotated as an ethylene responsive transcription factor and expressed from 32.70 to 22.46 at 0 h and 72 h, respectively. Similarly, the RTA joint_Locus_7247 which was annotated as encoding an AP2 domain transcription factor was expressed from 10.01 to 14.33 at 0 and 72 h, respectively. Both RTAs were grouped into cluster 6 by k-means analysis. RTAs with interesting annotations and expression trends related to stress factors are joint_Locus_19099 annotated as encoding a GST-30 which showed a decrease in expression from 60.98 at 0 h to 5.41 at 72 h, and joint_Locus_9459, annotated as a cytochrome P450 in maize, which showed an increase in expression from 1.82 at 0 h to 22.04 at 24 h.

### Candidate genes previously described in somatic embryogenesis

We performed an in-depth review of the literature to identify major candidate genes previously reported or suggested to be important for somatic embryogenesis in maize and other species, and using sequence similarity we identified orthologs in maize for genes identified in other species (Table 2).

**Table 2:** Somatic embryogenesis-related genes, National Center for Biotechnology Information (NCBI) accessions, and plant species where accession was previously characterized.

Gene name	NCBI gene accession number	NCBI protein accession number	Species	Reference
AGL15	U22528	AAA65653	Arabidopsis	[65]
AtLEC2	AF400124	AAL12005	Arabidopsis	[48], [76]
BnBBM1	AF317904	AAM33800	*B. napus*	[33], [34], [36], [37]
CLV1	U96879	AAB58929	Arabidopsis	[63]
ZAG1	L18924	AAA02933	Maize	[77]
ZAG2	L18925	AAA03024	Maize	[77]
ZmLEC1	AF410176	AAK95562	Maize	[47], [78]
ZMM2	L81162	AAB81103	Maize	[79]
ZmPIN1a	DQ836239	ABH09242	Maize	[54], [55]
ZmPIN1b	DQ836240	ABH09243	Maize	[54], [55]
ZmPIN1c	EU570251	ACB55418	Maize	[54]
ZmSERK1	AJ400868	CAC37640	Maize	[80], [81]
ZmSERK2	AJ400869	CAC37641	Maize	[80], [81]
ZmSERK3	AJ400870	CAC37642	Maize	[80]
ZmWUS1	AM234744	CAJ84136	Maize	[60]
ZmWUS2	AM234745	CAJ84137	Maize	[60]

Genes involved in stress responses previously suggested to be important in somatic embryogenesis include GST and germin like-proteins (GLP). Using gene accessions [23] and protein sequence similarity, we identified 15 maize GST genes, of which several showed an 8-fold or greater increase from 0 to 24 h (Table 3). In addition, one maize GLP gene GRMZM2G045809, annotated as ZmGLP2-1 [24], was up-regulated greater than 8-fold from 1.44 at 0 h to 251.27 FPKM at 72 h (Table 3). These stress response genes exhibiting a large fold change and increased expression from 0 to 24 h were grouped into k-means cluster 3.

**Table 3:** Expression of maize glutathione-S-transferase genes (ZmGST) and maize germin-like proteins (ZmGLP) in tissue culture.^a^

		FPKM						
Gene name	Annotated name	0 h	24 h	36 h	48 h	72 h	Fold change	Cluster number
ZmGST 8	GRMZM2G156877	0.63	25.66	32.87	33.53	24.51	8-fold	3
ZmGST 9	GRMZM2G126763	1.14	0.14	0.34	0.54	0.45	8-fold	2
ZmGST 10	GRMZM2G096153	21.22	17.45	18.86	23.83	24.42		6
ZmGST 11	GRMZM2G119499	1.02	0.92	2.81	7.00	7.15		4
ZmGST 12	GRMZM2G096269	1.04	0.84	2.43	4.76	2.32		4
ZmGST 13	GRMZM2G126781	0	0	0.22	0.65	0		
ZmGST 14	GRMZM2G175134	1.14	2.90	6.02	12.58	15.26	8-fold	3
ZmGST 15	GRMZM2G150474	0.18	3.25	3.85	3.66	2.64	8-fold	3
ZmGST 16	GRMZM5G895383	0	0.13	0.12	0.26	0.43		
ZmGST 18	GRMZM2G019090	7.05	121.54	206.88	175.17	105.79	8-fold	6
ZmGST 19	GRMZM2G335618	0.73	52.51	45.53	50.03	49.92	8-fold	3
ZmGST 20	GRMZM2G434541	1.93	16.64	10.82	8.91	4.71	8-fold	3
ZmGST 21	GRMZM2G428168	15.61	151.54	198.39	219.32	194.54	8-fold	6
ZmGST 22	GRMZM2G330635	59.24	208.94	140.95	136.98	118.47		6
ZmGST 23	GRMZM2G416632	5.56	211.88	200.78	209.11	143.91	8-fold	3
ZmGST 24	GRMZM2G032856	0.06	9.28	4.88	3.05	1.12	8-fold	1
ZmGST 25	GRMZM2G161905	0.91	51.28	15.15	6.36	1.75	8-fold	3
ZmGST 26	GRMZM2G363540	0.59	0.07	0	0	0	8-fold	
ZmGST 27	GRMZM2G077206	0.08	0.15	0.09	0.06	0		
ZmGST 28	GRMZM2G146475	7.88	16.79	13.13	15.44	11.86		6
ZmGST 29	GRMZM2G127789	0.45	0.70	0.59	0.61	0.44		2
ZmGST 30	GRMZM2G044383	41.73	49.57	30.16	15.25	3.37	8-fold	6
ZmGST 31	GRMZM2G475059	10.05	52.94	40.30	31.53	21.71		6
ZmGST 32	GRMZM2G041685	0	0.38	0.07	0.31	0.45		
ZmGST 33	GRMZM2G028821	0	0	1.28	6.88	11.82		
ZmGST 34	GRMZM2G145069	0	0	0	0	0		
ZmGST 34	GRMZM2G149182	0	0	0	0	0		
ZmGST 35	GRMZM2G161891	0	0.07	0.46	0.87	2.06		
ZmGST 37	GRMZM2G178079	4.09	15.62	17.86	18.05	20.88		6
ZmGST 38	GRMZM2G066369	0.21	0	0.26	0.54	0.29		
ZmGST 40	GRMZM2G054653	0.08	0.37	0.52	0.40	0.68	8-fold	2
ZmGST 41	GRMZM2G097989	26.40	26.00	22.70	32.69	34.13		6
ZmGST 42	GRMZM2G025190	2.10	197.29	133.85	90.71	66.98	8-fold	3
ZmGLP2-1	GRMZM2G045809	1.44	25.16	373.23	412.11	251.27	8-fold	3
ZmGLP3-1	AC190772.4_FG011	0	0.11	7.97	4.56	2.48		
ZmGLP3-2	GRMZM2G030772	0	0	6.04	5.85	1.73		
ZmGLP3-3	GRMZM2G149714	0	0.07	2.77	2.45	1.23		
ZmGLP3-16	GRMZM2G072965	0	0.15	0.24	0.49	0.17		
ZmGLP10-1	GRMZM2G178817	0	0.62	1.49	0.45	0.18		
ZmGLP10-2	GRMZM2G071390	0	0.64	0.59	0.34	0		
ZmGLP10-3	GRMZM2G049930	0	1.39	2.88	0.79	0.32		

a Fragments per kilobase of exon per million fragments mapped (FPKM) at 0, 24, 36, 48, and 72 h after placement on tissue culture initiation medium, genes with an 8-fold change in expression or greater as compared to the 0 h time point, and the assigned gene cluster designated by k-means analysis.

**Figure 3:**
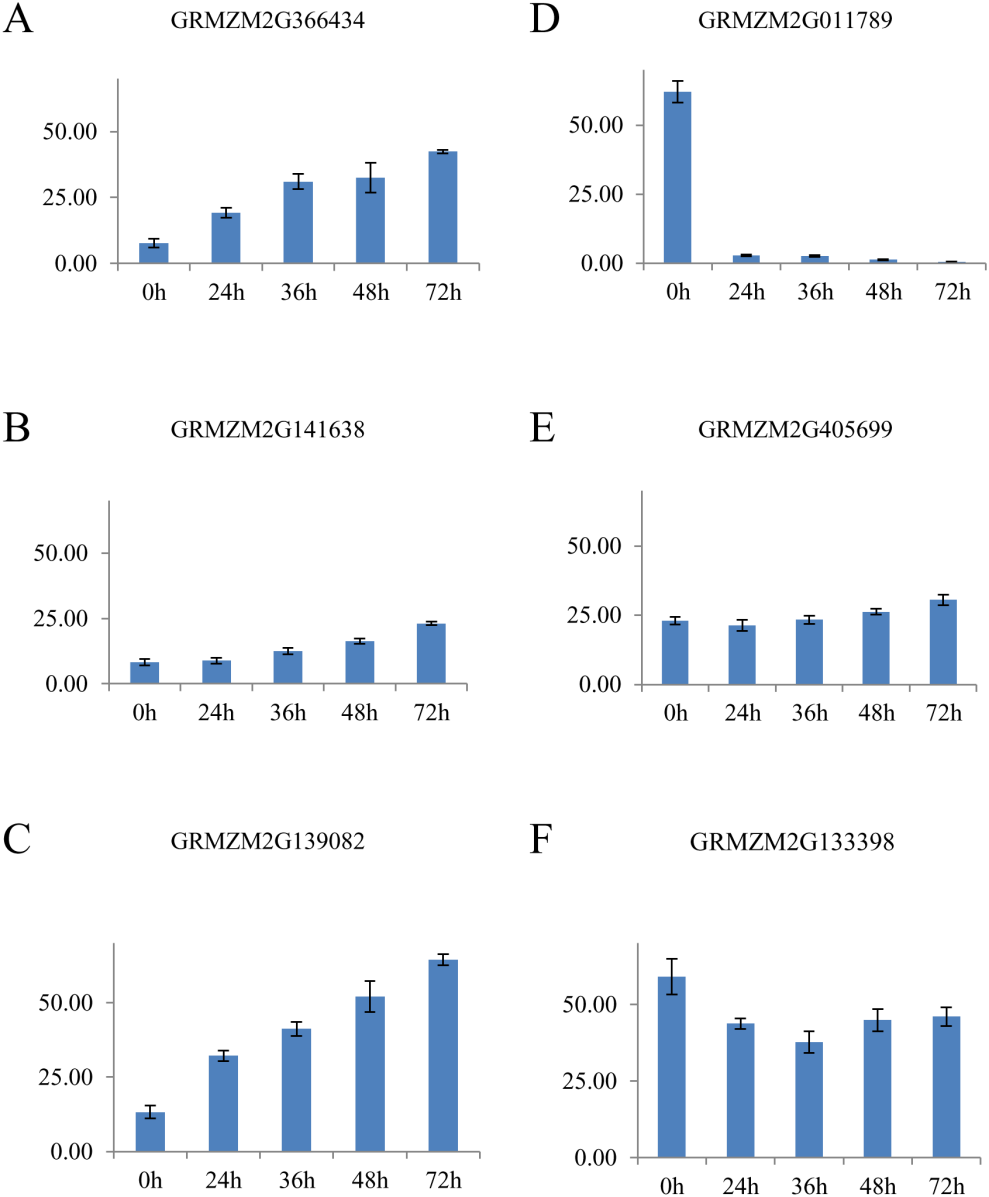
Gene expression of somatic embryogenesis genes involved in induction. Average values of fragments per kilobase of exon model per million fragments mapped (FPKM) of genes involved in the induction of somatic embryogenesis expressed in immature zygotic embryo explants of maize inbred line A188 at 0, 24, 36, 48, and 72 h after placement on culture initiation medium where (A), (B), and (C) are BBM-like maize genes with high sequence similarity to *Brassica napus* BABY BOOM (BnBBM1), (D) is maize LEAFY COTYLEDON1 (ZmLEC1), (E) is a maize gene with high sequence similarity to *Arabidopsis thaliana* LEAFY COTYLEDON2 (AtLEC2), and (F) is maize VIVIPARIOUS1 (VP1). Bars indicate average mean ± SE (n = 4 for 0, 24, 36, and 48 h include technical and biological replicates; n = 2 for 72 h include only technical replicates).

Genes involved in embryogenic pathway initiations include BABY BOOM (BBM) and LEAFY COTYLEDON (LEC) genes [7]. In this study, we highlight three maize genes that showed high sequence similarity to the highly conserved AP2 binding domain of *Brassica napus* BBM (BnBBM1, accession number AF317904). GRMZM2G366434, GRMZM2G141638, and GRMZM2G139082 are 91.2%, 92.5%, and 93.2% similar to the translated amino acid sequence of BnBBM1, respectively (Figure S1). GRMZM2G366434 showed a 4-fold up-regulation relative to 0 h at 36, 48 and 72 h (Figure 3A), GRMZM2G141638 also increased during this time course (Figure 3B), and GRMZM2G139082 increased over 4-fold from 0 to 72 h (Figure 3C). These maize BBM-like genes were grouped into cluster 3.

GRMZM2G011789, the maize ortholog to LEAFY COTYLEDON1 (ZmLEC1), was grouped into cluster 5 by k-means analysis. GRMZM2G011789 was expressed early initially (62.07 FPKM at 0 h) and then decreased dramatically to 2.90 at 24 h and 0.56 at 72 h (Figure 3D). Using sequence similarity, we found that maize gene GRMZM2G405699 is 47.4% similar in protein sequence to the Arabidopsis LEAFY COTYLEDON2 (AtLEC2, Figure S2) and 99.8% similar to the maize VIVIPARIOUS1 (VP1) gene GRMZM2G133398 (GenBank accession M60214). GRMZM2G405699 showed a moderate increase in expression during the time course of this study from 23.05 FPKM at 0 h to 30.50 FPKM at 72 h (Figure 3E), and GRMZM2G133398 (VP1) showed a different expression pattern with a moderate decrease from 59.04 FPKM at 0 h to 46.03 FPKM at 72 h (Figure 3F). GRMZM2G405699 and GRMZM2G133398 were grouped into k-means cluster 6.

SOMATIC EMBRYOGENESIS RECEPTOR-LIKE KINASE (SERK) genes are also important for embryogenic pathway initiation. In this study, expression of SERK1 (ZmSERK1, GRMZM5G870959) was minimal, ranging from 4.23 to 5.86 FPKM. Similarly, the orthologs to maize SERK2 (ZmSERK2, GRMZM2G115420) and the ortholog to maize SERK3 (ZmSERK3, GRMZM2G150024) showed very similar magnitudes in expression and trend increasing from about 15 to 20 FPKM. In our study, both ZmSERK2 and ZmSERK3 increased nearly 2-fold from 0 h to 24 h. Maize SERK genes were grouped into cluster 6.

**Figure 4:**
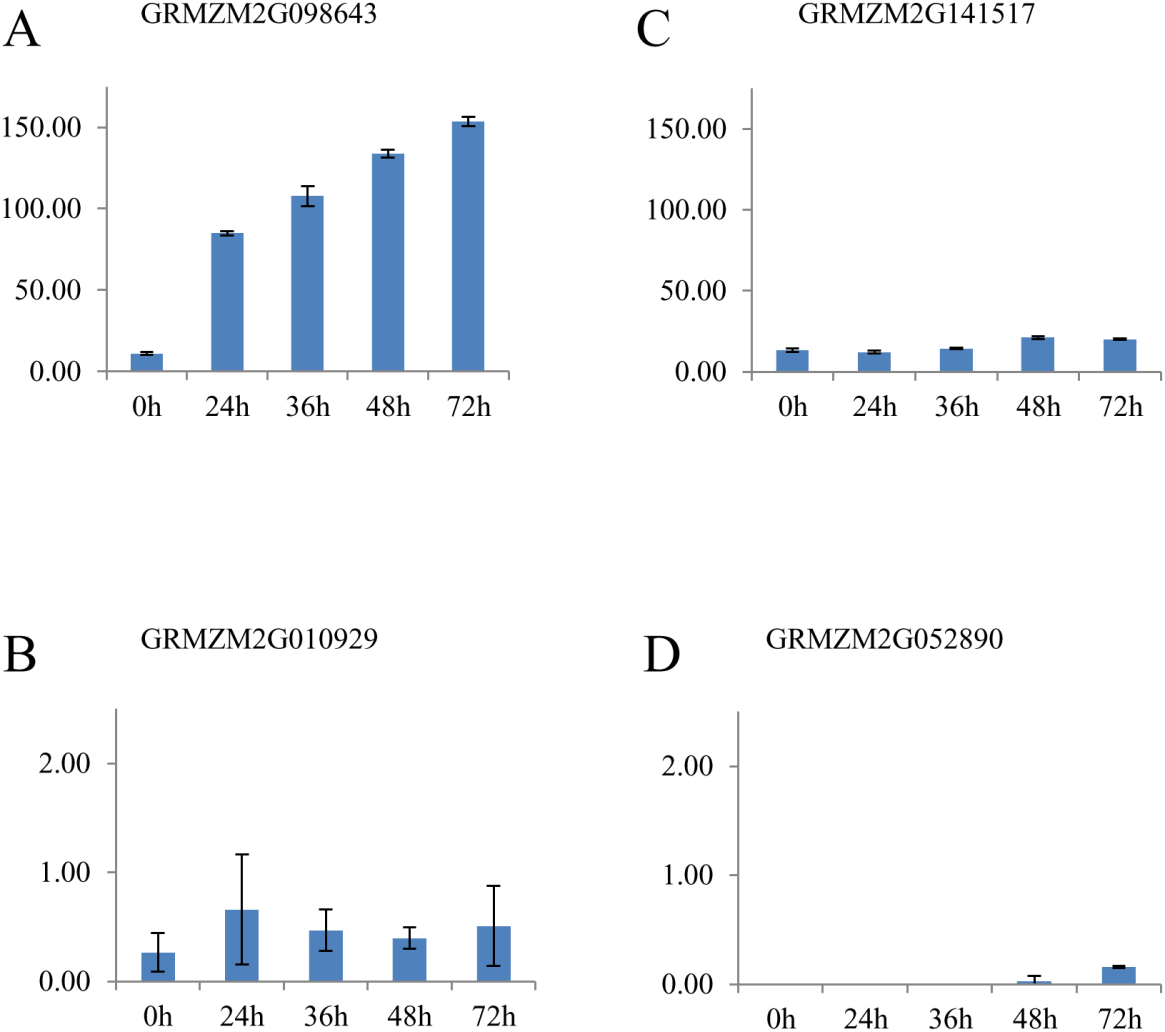
Gene expression of somatic embryogenesis genes involved in callus initiation and maintenance. Fragments per kilobase of exon model per million fragments mapped (FPKM) of maize genes associated with embryogenic callus induction expressed in immature zygotic embryos of maize inbred line A188 at 0, 24, 36, 48, and 72 h after placement on culture initiation medium where (A) is the maize ortholog to PINFORMED1 (ZmPIN1a), (B) is the maize ortholog to WUSCHEL (ZmWUS1), (C) is maize gene with high sequence similarity to CLAVATA (CLV1) and (D) is a maize ortholog to AGAMOUS (ZAG1). Bars indicate average mean ± SE (n = 4 for 0, 24, 36, and 48 h include technical and biological replicates; n = 2 for 72 h include only technical replicates).

PIN1 is involved in auxin transport [25]. The maize PINFORMED1 (PIN1) gene, (ZmPIN1a, GRMZM2G098643) displayed up-regulation with FPKM values of 11.02 at 0 h to 153.59 at 72 h (Figure 4A), and additional orthologs to maize PINFORMED1 (ZmPIN1b, GRMZM2G074267) and (ZmPIN1c, GRMZM2G149184) also increased in expression. ZmPIN1a and ZmPIN1b were grouped into cluster 6; ZmPIN1c was grouped into cluster 3 (Figure 2).

Known genes involved in embryo formation and development include WUSCHEL, CLAVATA, AGAMOUS and WOX genes. ZmWUS1 (GRMZM2G010929) was minimally expressed, not exceeding 1 FPKM during this time course (Figure 4B), and ZmWUS2 (GRMZM2G028622) was not expressed in any sample. GRMZM2G14151 has high sequence similarity to the CLAVATA (CLV1) gene in Arabidopsis (Figure S3) and increased in expression from 13.14 FPKM at 0 h to 20.06 FPKM at 72 h (Figure 4C). GRMZM2G14151 was grouped into k-means cluster 6. Maize genes that are orthologs to AGAMOUS, which include ZMM2 (GRMZM2G359952), ZAG1 (GRMZM2G052890), and ZAG2 (GRMZM2G160687), showed minimal expression during the time course of this study (Figure 4D and Table 2). A BLAST search for AGL15 revealed a number of maize genes with high sequence similarity. For example, ZmMADS69 (GRMZM2G171650), ZmMADS52 (GRMZM2G446426), and ZmMADS73 (GRMZM2G046885) show 67.74%, 64.41%, and 42.11% sequence similarity to the AGL15 amino acid sequence in Arabidopsis. These MADS box transcription factors were grouped into k-means cluster 6 and were moderately expressed throughout the time course, with FPKM values greater than 10 at every time point (Figure S4A–C). One maize gene, ZmMADS11 (GRMZM2G139073), has 45.75% sequence similarity to AGL15. ZmMADS11 was grouped into k-means cluster 3 and was shown to have an 8-fold expression change at each time point compared to 0 h (Figure S4D). We also examined the expression of maize WUSCHEL-related homeobox domain (WOX) genes and found ZmWOX2A (GRMZM2G108933), ZmWOX5A (GRMZM2G478396), ZmWOX5B (GRMZM2G116063), and ZmWOX11/12B (GRMZM2G314064) showed an 8-fold increase in expression after placement of immature embryos into the tissue culture environment and grouped into k-means clusters 1, 2, and 3 while other maize WOX genes were grouped into clusters 5 and 6 (Table 4).

**Table 4:** Expression of WUSCHEL-related maize WOX genes in tissue culture.^a^

			FPKM						
Gene name	Annotated name	Sequence similarity(%)	0 h	24 h	36 h	48 h	72 h	Fold change	Cluster number
ZmWOX2A	GRMZM2G108933	100	0.70	0.34	0.08	0.04	0.11	8-fold	2
ZmWOX2B	GRMZM2G339751	100	0.18	0.68	0.15	0	0		
ZmWOX3A	GRMZM2G122537	100	0.23	0.98	0.32	0.53	0.39		2
ZmWOX3B	GRMZM2G069028	85.71	1.50	1.10	0.52	0.22	0.79		5
ZmWOX3B	GRMZM2G140083	84.81	0	0	0	0	0		
ZmWOX5A	GRMZM2G478396	96.72	0	4.51	9.06	6.20	6.61	8-fold	
ZmWOX5B	GRMZM2G116063	100	0.06	2.17	1.91	4.53	6.27	8-fold	1
ZmWOX9A	GRMZM2G133972	100	0.49	0.55	0.19	0.07	0		
ZmWOX9B	GRMZM2G031882	100	5.95	3.07	2.91	2.08	1.57		6
ZmWOX9C	GRMZM2G409881	100	5.53	3.01	4.86	3.42	2.85		6
ZmWOX11/12B	GRMZM2G314064	98.46	2.51	11.29	15.50	17.96	14.80	8-fold	3
ZmWOX13A	GRMZM2G038252	100	0	0	0	0	0		
ZmWOX13A	GRMZM2G069274	100	0	0	0	0	0		
ZmWOX13B	GRMZM5G805026	100	5.86	5.46	6.21	7.46	6.05		6
KNOTTED1	GRMZM2G017087	100	20.67	15.33	11.52	10.66	10.94		6

a Fragments per kilobase of exon per million fragments mapped (FPKM) at 0, 24, 36, 48, and 72 h after placement on tissue culture initiation medium, genes with an 8-fold change in expression or greater as compared to the 0 h time point, and the assigned gene cluster designated by k-means analysis.

## Discussion

**Figure 5:**
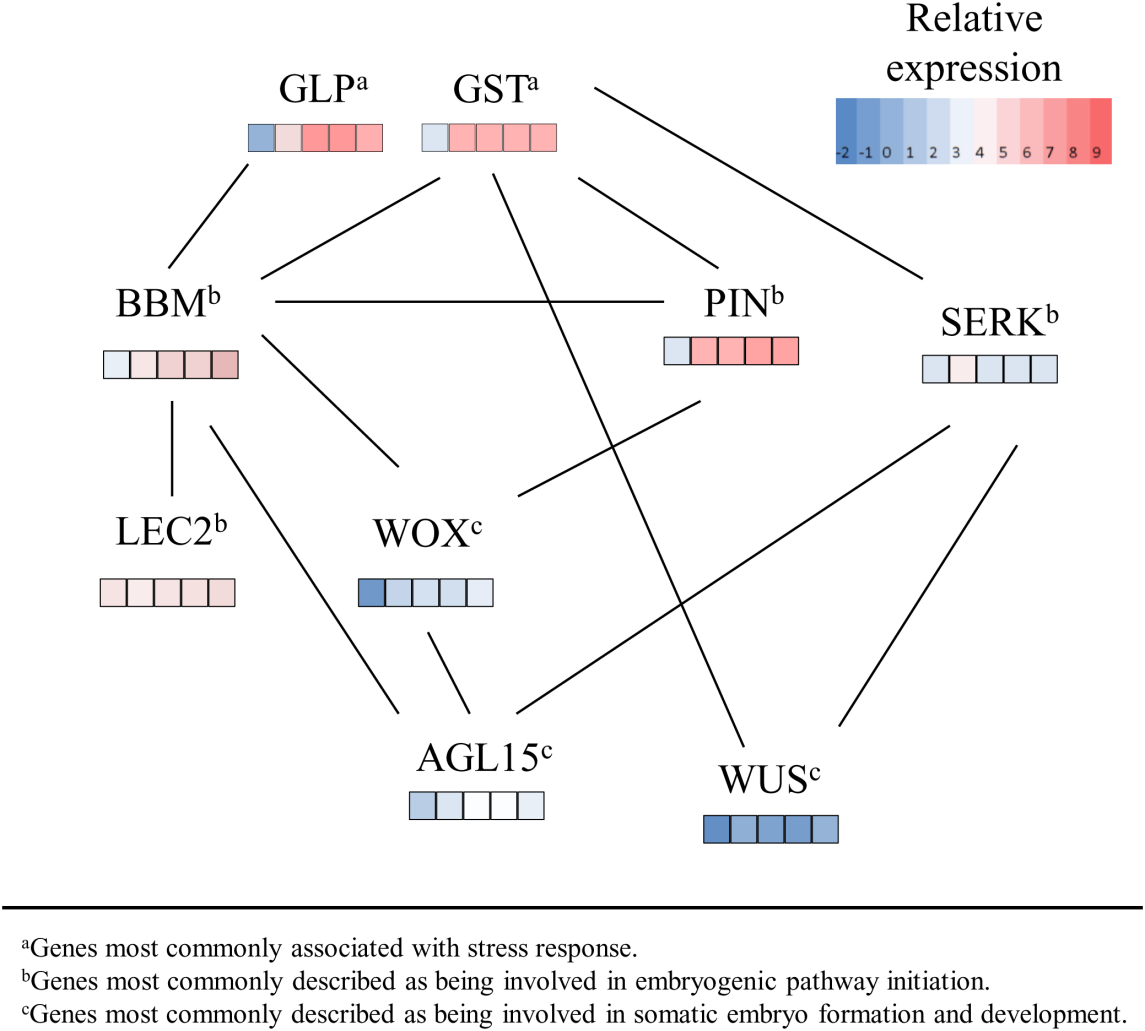
Relative expression of coexpressed somatic embryogenesis-related genes. A proposed model of somatic embryogenesis-related gene expression networks as determined by coexpression (solid lines) with a correlation coefficient greater or equal to 0.9 between genes expressed during the early stages of somatic embryogenesis. Relative expression of transcripts detected in immature zygotic embryo explant tissues in tissue culture were detected in the inbred line A188 and reported as the average log2 expression displayed by color coded values as depicted by the figure legend for each gene. The first left most box under each gene name is the average log2 transformation of fragments per kilobase of exon model per million fragments mapped (FPKM) at 0 h, the second box at 24 h, the third at 36 h, the fourth at 48 h, and the fifth right most box is the average FPKM 72 h. Glutathione-S-transferases (GSTs) and germin-like proteins (GLPs) are stress response genes that are triggered in early somatic embryogenesis. BABY BOOM (BBM), an APETALA-like ethylene-responsive element transcription factor, and LEAFY COTYLEDON2 (LEC2), a B3 domain transcription factor, promote somatic embryogenesis. PINFORMED (PIN) genes mediate auxin transport and establish essential endogenous auxin concentrations in the cell. SOMATIC EMBRYOGENESIS RECEPTOR LIKE KINASES (SERK) genes are also involved in somatic embryogenesis and hormone metabolism. WUSCHEL (WUS), a homeodomain transcription factor, regulates stem cell fate during embryo formation and development. AGAMOUS like-15 (AGL15), a MADS box transcription factor, also promotes somatic embryo formation and is also involved in meristem development. WUSCHEL-related homeobox domain (WOX) genes have also been detected during somatic embryogenesis in embryogenic genotypes but not in non-embryogenic genotypes.

Somatic embryogenesis-related genes have been extensively characterized in Arabidopsis; however, relatively few have been evaluated in maize. Using transcriptome data of maize in embryogenic tissue culture initiation, this study provides an in-depth look at the major candidate genes discussed in previous reviews and research studies on somatic embryogenesis. Moreover, we propose a model (Figure 5) based on coordinated expression (Table S8) of somatic embryogenesis-related genes highlighted in this study and their relative expression in early embryogenic tissue culture response.

### Genes associated with stress response in tissue culture

Our observations support previous reviews on the transition to somatic embryogenesis, with our whole transcriptome data showing a large number of genes expressed during the early stages of somatic embryogenesis from 0 h to 24 h in tissue culture. Gene enrichment analysis of genes clustered based on k-means, and of genes grouped by large fold changes when compared to the control time point 0 h, supported major biological functions suggested in previous studies as important for somatic embryogenesis such as stress response, transmembrane transport, and hormone metabolism [6], [8], [9], [21]. Genes with a large fold change and genes grouped in cluster 3 in this study include cytochrome P450, UDP-glucosyl, and glucoronyl transferases. In another study involving an embryogenic maize line from China, genes differentially expressed in the early stages of embryogenesis were also related to stress where metabolism of xenobiotics by cytochrome P450 was identified as one of the most significant pathways by enrichment analysis of differentially expressed genes in samples grown 1–5 days after tissue culture [26].

In this study, we identified two maize genes, WIP1 (GRMZM2G156632) and chitinase A1 (GRMZM2G051943) which were up-regulated over 1500-fold from 0 h to 24 h. These genes have been previously described in plant defense and stress response [27], [28], but have not, until now, been associated with tissue culture response in maize. WIP1 has previously been characterized as a defense gene based on its involvement in hypersensitive defense response [28]. Chitinase proteins have been suggested to promote somatic embryogenesis [9] since one study in carrot showed that a non-embryogenic mutant line was triggered to produce somatic embryos after the addition of chitinase proteins in the tissue culture medium [29].

GSTs are a family of genes also involved in plant defense [30] and we observed 15 out of the 33 maize GST genes with large fold expression changes during early somatic embryogenesis (Table 3). It has been suggested that some GSTs may function in tissue dedifferentiation by affecting the cell’s redox status by changing endogenous levels of important plant growth hormones such as auxin [6]. GSTs were also detected in chicory during somatic embryogenesis in callus cultures initiated with leaf tissue [30], and GSTs were also expressed in response to auxin treatment in *Cyclamen persiucum* tissue culture with an initial up-regulation during the first 4 hours followed by down-regulation at 72 h [31]. In this study, GST genes were found to be coexpressed with BBM, WUS, PIN, and SERK genes (Figure 5, Table S8). We also detected one maize GLP gene (GRMZM2G045809) with a large fold change in expression at 72 h (Table 3). Moreover, this GLP gene was shown to be coexpressed with the BBM transcription factor (Figure 5). GLPs are proteins that also affect the plant redox status and are involved in developmental regulation. In wheat embryogenic callus cultures, GLPs were detected as early as 2 to 72 hours after plating explant tissues in culture [32]. GLPs are typically detected in embryogenic tissues, but not in non-embryogenic tissues. GLPs with superoxide dismutase activity promote the production of hydrogen peroxide (H_2_O_2_), a type of oxidative stress. It has been suggested that the H_2_O_2_ produced may serve as a secondary signaling molecule acting to promote somatic embryogenesis [8], [9].

### Genes involved in embryogenic pathway initiation

Embryogenic pathway initiation is marked when somatic cells acquire embryogenic competence and proliferate as embryogenic cells capable of forming somatic embryos [8]. One gene that has been attributed to initiation of somatic embryogenesis across plant species is BBM. BBM genes highlighted in this study were found to be coexpressed with GLP, GST, PIN, WOX, LEC2, and AGL15 genes (Figure 5). BBM was first discovered in investigations of *Brassica napus* microsporogenesis by subtractive hybridization [33]. The gene was consistently expressed only in embryogenic microspore cultures. Sequence analysis showed that BBM has two unique binding domains: an APETALA-like AP2 binding domain and an ethylene-responsive element binding factor, both characteristic of functioning in plant hormone signaling and regulation [33]. Overexpression of BBM in Arabidopsis and *B. napus* led to the induction of somatic embryogenesis and regeneration ability without the addition of exogenous plant hormones [33]. This observation suggested that BBM acts as a stimulator of plant hormone production, triggering signaling pathways important for somatic embryogenesis [33], [34]. Overexpression of BBM-induced embryo formation enhanced regeneration ability in Chinese white poplar [35] and tobacco, [36] and improved transformation efficiency in sweet pepper [37]. In another study focused on transforming artificial chromosomes into maize, the shuttle vector used contained a BBM homolog called ZmODP2 to promote cell division and callus growth after transformation [38]. Researchers suggested that the presence of this construct improved transformation efficiency in maize tissue culture by 20–50%. In our study, we identified three maize genes GRMZM2G366434, GRMZM2G141638, and GRMZM2G139082 with high sequence similarity to BnBBM1 and which contain the conserved and unique AP2 binding domain (Figure S1). These maize genes were also shown to increase in expression during early somatic embryogenesis (Figure 3) in this study. When we compared these maize gene expression trends to transcripts detected in the maize B73 gene atlas [39], expression was not at all detected (0 FPKM) in whole seeds or endosperm at 10, 12, 14, and 16 days after pollination. Expression was, however, detected in zygotic embryos 16 days after pollination, in germinating seed, in the primary root and in V3 stem and shoot apical meristem [40].

Another important group of genes involved in embryogenic pathway initiation are the LEC genes. LECs are transcription factors identified in studies of zygotic embryogenesis in plants that have been proposed to be important for somatic embryogenesis [7], [41]. Mutational analysis of LEC genes showed their function in early zygotic embryogenesis, specifically, to maintain suspensor cell fate and specify cotyledon identity [42]. LEC genes play an important regulatory role directly interacting with hormone response genes [43], [44]. AtLEC1 was cloned and ectopically expressed in transgenic Arabidopsis seeds, showing its essential role in germination and embryonic organ identity [45]. AtLEC1 in Arabidopsis in tissue culture was also shown to be differentially expressed in embryogenic compared to non-embryogenic samples [46]. One study on the highly embryogenic maize hybrid HiII, ZmLEC1 transcription in somatic embryos showed a high initial expression and then a decrease in expression during early development of [47]. We found a similar result in our study, where ZmLEC1 (GRMZM2G011789) decreased in expression over 20 fold during the first 24 hours in tissue culture. In contrast, the expression of a maize gene similar to AtLEC2 (GRMZM2G405699) based on high sequence similarity (Figure S2) increased steadily in this study (Figure 3), was grouped into k-means cluster 6 (Table S6), and is coexpressed with BBM (Figure 5). The role for AtLEC2 in Arabidopsis zygotic embryogenesis to induce somatic embryos by activating auxin responsive genes was proven by ectopic expression [48], [49]. AtLEC2 is nearly identical to VP1 in Arabidopsis [7], [49] and in this study, we found that the maize gene GRMZM2G405699, which is most similar to AtLEC2, is also highly similar to maize VP1 (GRMZM2G133398). Both AtLEC2 and VP1 genes share the same class of unique B3 domains. One study involving gene expression analysis on T-DNA insertion lines in Arabidopsis suggested a role for VP1-like genes in recruiting chromatin-remodeling factors that can either activate or repress LEC1-like activity during seed development [50]. Moreover, it has been suggested that this complex network involving LEC1 and LEC2 genes in seed development can up-regulate important transcription factors such as BBM during early zygotic embryogenesis in Arabidopsis [44]. Our study showed GRMZM2G405699 coexpressed with maize BBM-like genes during early somatic embryogenesis (Figure 5).

PIN1 genes encode influx and efflux carrier proteins that mediate auxin transport in early zygotic embryo development [51]. In this study, PIN1 was coexpressed with GST, BBM, and WOX genes (Figure 5). In Arabidopsis, PIN genes, are essential for embryonic stem cell growth [52] and are expressed in early proembryonic development [53]. In maize, PIN1 genes also play a role in auxin transport and tissue differentiation during zygotic embryogenesis [54]. The ZmPIN1a gene was highly expressed in this study (Figure 4A), and increased dramatically from the 24 to 72 h time point. A study using *in situ* hybridization of ZmPIN1a and ZmPIN1b showed transcript abundance and protein localization of PIN proteins in maize kernels, endosperm and embryo [54], [55]. The authors also suggested a role for PIN1 in maize development during zygotic embryogenesis in mediating polar auxin transport and patterning during development [25], [54]. In tissue culture, an important step in establishing embryogenic patterning in embryos is apical-basal rearrangement [51]. Our observations also show that PIN1 genes are expressed during tissue culture response in early somatic embryogenesis.

### Genes involved in somatic embryo formation and development

There is evidence that genes involved in meristem formation are also important in somatic embryo formation. For example, WUS is a homeodomain transcription factor involved in shoot and floral meristem development specifically as a regulator of stem cell fate and organ identity [56]. WUS expression has been detected in a small group of cells described as the organizing center of meristematic tissue. This organizing center is localized underneath a larger mass of stem cells [52], [57], [58]. WUS has an important role in regulating and activating pluripotent stem cells by promoting proliferation genes and repressing developmental regulators [59]. While, it has been shown in Arabidopsis that PIN1-mediated auxin transport directly induces WUS expression in early somatic embryogenesis [52], in our study, ZmWUS genes were minimally expressed, however, were coexpressed with GST and SERK genes (Figure 5). We hypothesize that the developmental window highlighted in this study may have captured a time when the organizing center was just initiating in development and that transcripts detected represented few cells showing WUS activity during the early stages of stem cell development. In addition, it is plausible that suitable endogenous auxin concentrations were just beginning to establish. Over time, more cells either localized in or on the organizing center would also display WUS transcriptional activity.

Detailed analysis of the expression pattern of WUS orthologs in maize and rice showed that WUS genes in higher plants did not mimic expression localized in the organizing center as it did in Arabidopsis implying a major modification in plant evolution [60]. Our findings showed that WUS genes had minimal to no expression in early embryogenesis in tissue culture but some maize WOX genes increased in expression over 8-fold. WOX genes were coexpressed with BBM, PIN and AGL15 (Figure 5). WOX expression was detected in somatic embryogenesis in other plants where efficient embryogenic callus cultures are also genotype-dependent [61]. In our study, we highlight a number of maize WOX genes with differential expression compared to time point 0 h (Table 4) of which ZmWOX2A, ZmWOX5A/5B, and ZmWOX11/12B showed an 8-fold change in expression. Some examples of WOX genes in tissue culture in other plants include, WOX2 associated with somatic embryogenesis in conifer tissue culture [62] and WOX11 in grapes detected in embryogenic versus non-embryogenic cultivars *in vitro* [61].

Expression of a CLV1-like gene (GRMZM2G141517), however, showed a steady increase in this study (Figure 4C). CLV1 is a receptor-like kinase also involved in shoot and floral meristem development [63] and acts upstream of WUS. CLV1 represses WUS activity by interacting in a regulatory loop with WUS to promote callus initiation and maintenance [64]. Capturing expression of these genes at later time points in tissue culture would provide insight on transcriptional activity between the regulatory loop between WUS and CLV1 in maize.

Another meristem-related gene discovered in Arabidopsis is AGAMOUS, a MADS box transcription factor involved in flower development and organ differentiation [65]. AGAMOUS had been shown to interact directly with WUS also by repressing its expression in floral stem cells [58], [66]. Similar to ZmWUS, genes highly similar to AGAMOUS, such as ZAG1, ZAG2 and ZMM2 were minimally expressed in this study. However, we did observe differential expression relative to 0 h for an AGL15-like gene (GRMZM2G139073) (Figure S4D). In addition, GLP, PIN, WOX and BBM genes were coexpressed with GRMZM2G139073 (Figure 5, Table S8). AGL15 in *B. napus* and Arabidopsis embryos [65] have been shown to be preferentially localized in embryonic tissues [67]. Additionally, AGL15 was shown to promote somatic embryo development in Arabidopsis and soybeans [68]. Studies also suggest that AGL15 in Arabidopsis interacts with LEC2 directly [43], [69] and, immunoprecipitation and time-of-flight mass spectrometry revealed AGL15 was included in the SERK1 complex *in vivo* [70]. To date, there have been no studies of AGL15–like genes expressed in maize somatic embryos that have been reported. From studies on AGL15 in Arabidopsis in promoting somatic embryogenesis and interacting with LEC2 and SERK1, we hypothesize that maize AGL15-like genes may also be important for callus initiation and maintenance.

## Conclusion

Deciphering the underlying genetic mechanisms controlling somatic embryogenesis in tissue culture is important for improving our understanding of the basic processes involved in somatic embryo formation, and in the development of embryogenic tissue culture systems that are less genotype dependent. Although few major genes that promote somatic embryogenesis in Arabidopsis and other plants species have been described, even fewer genes have been studied and their expression revealed in the context of the whole transcriptome in tissue cultures of maize. In this study, we highlighted the expression of maize genes with high sequence similarity to BBM, LEC2, CLV1, and AGL15, and maize SERK and PIN genes, and discussed their potential role in somatic embryogenesis. Many of the somatic embryogenesis related genes analyzed in this study fall into a k-means clusters 3 with an expression trend towards an initial large up-regulation and a second cluster number 6, with genes that are moderately to highly expressed throughout the targeted developmental. However, clusters 4 and 5 also show interesting expression trends that could be important for further studies due to their large up- and down-regulation expression trends, respectively. In this investigation, we also highlighted maize gene families, mainly GST, GLP, and WOX genes and identified specific genes within gene families with altered expression. A number of specific genes discussed in this study could be potential candidates for further testing regarding their importance and contribution to embryogenesis in tissue culture in maize.

Whole transcriptome profiling during the very early stages in the initiation of somatic embryogenesis in culture of the highly embryogenic, regenerable maize genotype, A188, now provides new information on the expression of somatic embryogenesis-related genes in maize. By studying the whole transcriptome during a specific developmental window, we were able to provide data on transcripts detected for major genes previously described with a role in embryogenesis. This information can be utilized to help us better understand major gene functions and expression networks involved in the induction of somatic embryogenesis in culture. Investigations involving fine-mapping and identification of specific genes in maize that confer regeneration ability could build on the findings reported here to further enhance our understanding of which many genes expressed in concert are possible key factors underlying the genotype dependent nature of tissue culture phenotypes. In the same way, a study involving the analysis of the whole transcriptome of isogenic lines differing in their ability to produce embryogenic, regenerable cultures, and their representative transcripts that are not mapped to the reference genome, could also add to identifying causal genes, providing a deeper understanding of the somatic embryogenesis-related genes we described here, and allow determination of their level of significance in the process. Improving our understanding of the biological processes and the genetic mechanisms that confer efficient tissue culture response such as somatic embryogenesis *in vitro* will help crop improvement strategies and functional genomics testing that is necessary to increase agricultural productivity in a changing global agricultural landscape.

## Materials and Methods

### Plant material and tissue culture initiation

Field grown donor plants were grown at the West Madison Agricultural Research Station (Madison, WI). Immature maize embryos from two plants of the maize inbred line A188 were isolated and cultured as previously described [71] with minor modifications. Briefly, ten days after pollination, 125 immature embryos (1.0–1.2 mm from scutellar tip to base) from each of two maize ears were harvested, aseptically dissected from kernels, and then placed onto culture initiation medium by placing embryos axis side down (scutellum side up) on modified N6 tissue culture medium [72]. The medium was prepared with N6-basal salts [72] at 3.98 g/L (PhytoTechnolgies Lab, product number M524), 2 mL/L of 1 mg/mL 2, 4-D stock, 2.875 g/L L-proline, 30 g/L sucrose, 3.5 g/L gelzan, pH to 5.8. After autoclaving, filter sterilized N6 vitamins stock (1,000x solution) and silver nitrate stock solution prepared as per protocol [71] were added. Prior to embryo isolation, ears were surface sterilized in a 50% commercial bleach (8.25% sodium hypochlorite) solution with a drop of Tween 20, and then rinsed 3 times in sterile, deionized water. A total of 210 embryos from each of two donor plants, or two biological replicates, were used for this study. Ten to 25 embryos were harvested for the each of 0, 24, 36, and 48 h time points. Ten embryos from only one plant, or one biological sample, were harvested for the 72 h time point. For the first time point (0 h), the embryos were aseptically dissected from kernels and immediately placed into liquid nitrogen without placement on culture medium. For subsequent time points at 24 h, 36 h, 48 h, and 72 h after plating, embryos were aseptically isolated and placed onto culture initiation medium.

### RNA-seq Library Construction and Sequencing

RNA was extracted using the Invitrogen TRIzol reagent according to the manufacturer’s instructions (Invitrogen, http://www.invitrogen.com). Samples were processed using the RNeasy MinElute Cleanup kit (Qiagen, http://www.qiagen.com). RNA quality was assessed using the Agilent RNA 6000 Pico Kit Bioanalyzer prior to preparation of the sequence library. Approximately 5 µg of total RNA was processed for mRNA isolation, fragmented, converted to cDNA, and PCR amplified according to the Illumina TruSeq RNA Sample Prep Kit as per the provided protocol, and sequenced on an Illumina HiSeq 2000 (San Diego, CA) at the University of Wisconsin Biotechnology Center (Madison, WI). Two technical sequencing replicates were conducted for each of the two biological sample collections for the 0, 24, 36, and 48 h time points and one biological sample for the 72 h time point, each with 101 nucleotide single-end reads. Sequences are available in the Sequence Read Archive at the National Center for Biotechnology Information (BioProject accession number PRJNA242658). Sequence quality for each sample was evaluated using the FastQC software (http://www.bioinformatics.bbsrc.ac.uk/projects/fastqc) and all samples passed quality control analysis. For subsequent analyses, FPKM values from the two technical sequencing replicates were averaged to represent transcript abundance for each time point 0, 23, 36, and 48 h. FPKM values from two technical sequencing replicates were averaged from one biological sample for time point 72 h.

### Data Analysis

To quantify transcript abundance, sequence reads for each sample were mapped to the maize v2 pseudomolecules (AGPv2; http://ftp.maizesequence.org) [17] and 8,681 non-RTAs that were assembled using RNA-seq reads from 503 diverse maize inbred lines [18]. Mapping was performed using Bowtie version 0.12.7 [73] and TopHat version 1.4.1 [74] with a minimum and maximum intron length of 5 bp and 60,000 bp respectively and the no-novel-indels option. All other parameters were set to the default values. Normalized gene expression levels were determined using Cufflinks version 1.3.0 [19] setting a maximum intron size of 60,000 bp, the version 5b annotation (http://ftp.maizesequence.org) as the reference annotation, and the AGPv2 fasta sequences for the bias detection and correction algorithm. All other parameters were set to the default values. Pearson's correlation of transcript abundance estimates were measured between biological replicates. Transcripts for samples for 0, 24, 36, and 48 h time points were averaged between the two biological replicate samples while transcripts for the 72 h time point represented transcripts detected in only one biological sample.

K-means clusters were determined using uncentered Pearson’s correlation coefficients in DNA Star ArrayStar version 5.1.0 build 114 allowing 6 clusters and 100 iterations. Only genes with an FPKM value greater than zero at any given time point were included. For an analysis of differential gene expression, each time point was compared to the control time at 0 h. A threshold for differential expression of greater than 8-fold for raw FPKM values was used. In order to include genes that may have not been expressed at any given time point but then showed expression at other time points, we included genes with a sum of 2 FPKM or greater in the differential gene expression analysis. Raw values were log2 transformed and visualized on a scatter plot in DNA Star ArrayStar version 5.1.0 build 114. In order to determine coexpression of selected genes, an analysis was done in the R using the xtable statistical computing package version 3.0.2 to calculate the Pearson correlation where the minimum coefficient was set to a threshold of 0.75. In the discussion highlighting the coexpression of specific somatic embryogenesis-related genes, the threshold was set to 0.90.

Gene ontology enrichment analysis was conducted in the PlantGSEA database (http://structuralbiology.cau.edu.cn/PlantGSEA/index.php) [75] to describe groups of genes in specific clusters or groups of genes differentially expressed with large fold expression changes in different time point comparisons. Enrichment analysis determined maize gene sets that characterized each group as determined by statistical analysis. Fisher’s exact test took into account the number of genes in the group query, the total number of genes in a gene set, and the number of overlapping genes. A multiple test false discovery rate correction using the Yekutieli method was set to a cutoff *P-*value at 0.05. Additional annotations were determined by MapMan genome release for *Zea mays* based on B73 5b filtered gene sets (http://mapman.gabipd.org/) [22].

### Maize sequence similarity

Maize genes with high sequence similarity to somatic embryogenesis related genes were determined by comparing the maize 5b.60 protein sequences to the protein sequence of previously cloned and characterized genes using BLASTP in the MaizeGDB BLAST POPcorn Project Portal (http://popcorn.maizegdb.org/main/index.php). Input parameters were set to an e-value cutoff of 1e-4 and the maximum number of hits was set to 500. Maize genes with a percent identity greater than or equal to 50% were analyzed for the presence of the conserved binding domain or other features specific to the gene of interest in the National Center for Biotechnology Information (NCBI) Batch Web CD-Search Tool (http://www.ncbi.nlm.nih.gov/Structure/bwrpsb/bwrpsb.cgi) and NCBI Conserved Domains CD-Search tool (http://www.ncbi.nlm.nih.gov/Structure/cdd/wrpsb.cgi) to determine genes with the best match. Sequence similarity reported in this study by pairwise alignment was done in LALIGN (http://embnet.vital-it.ch/software/LALIGN_form.html) as the percent identity by local or global alignment.

## Supporting Information

Figure S1 **Sequence alignment to determine sequence similarity of BABY BOOM in maize.** Multiple sequence alignment of the conserved 147 amino acid sequence of BABY BOOM1 (gene accession AF317904) and three maize annotated proteins with high sequence similarity: GRMZM2G366434_P01, GRMZM2G141638_P01, and GRMZM2G139082_P02.
(TIF)

Figure S2 **Sequence alignment to determine sequence similarity of LEAFY COTYELODN2 in maize.** Sequence alignment between LEAFY COTYELODN2 in Arabidopsis (gene accession AF400124) and maize protein GRMZM2G405699_P01.
(TIF)

Figure S3 **Sequence alignment to determine sequence similarity of CLAVATA in maize.** Sequence alignment of the 191 amino acid translated sequence representing the catalytic domain of protein kinases superfamily of CLAVATA (CLV1) in Arabidopsis (gene accession U96879) and the maize protein GRMZM2G141517_P01.
(TIF)

Figure S4 **Gene expression trends in early somatic embryogenesis of maize genes that are similar to AGL15.** Fragments per kilobase of exon model per million fragments mapped (FPKM) of maize genes with high sequence similarity to Arabidopsis gene AGL15 (gene accession U22528) associated with embryogenic callus induction expressed in immature zygotic embryos of maize inbred line A188 at 0, 24, 36, 48, and 72 h after placement on culture initiation medium. Genes shown include (A) ZmMADS69 (GRMZM2G171650), (B) ZmMADS52 (GRMZM2G446426), (C) ZmMADS73 (GRMZM2G046885), and (D) SILKY1 (GRMZM2G139073). (n = 4 for 0, 24, 36, and 48 h include technical and biological replicates; n = 2 for 72 h include only technical replicates).
(TIF)

Table S1 **Summary data on transcripts detected.** A summary of the number of reads mapped for each sample of immature zygotic embryos from two ears of maize inbred line A188 collected at 0, 24, 36, 48 h and one ear collected at 72 h after placement on tissue culture initiation medium.
(XLSX)

Table S2 **Correlation of transcript abundance between biological replicates across time.** Pearson's correlation of transcript abundance estimates measured as fragments per kilobase of exon model per million fragments mapped (FPKM) between samples of immature zygotic embryo explants from two maize ears of inbred line A188 collected at 0, 24, 36, 48, and 72 h after placement on tissue culture initiation medium.
(XLSX)

Table S3 **Gene expression of reference genes and representative transcript assemblies (RTA) detected in early somatic embryogenesis.** Fragments per kilobase of exon model per million fragments mapped (FPKM) of gene transcripts detected in immature zygotic embryo explants of maize in inbred line A188 at 0, 24, 36, 48, and 72 h after placement on tissue culture initiation medium averaged across two biological replicates and gene annotations based on MapMan 5b filtered gene set gene names and annotations for the joint loci representative transcript assemblies (RTAs).
(XLSX)

Table S4 **Genes differentially expressed at each time point compared to control and expression trend.** Fragments per kilobase of exon model per million fragments mapped (FPKM) values of genes or representative transcript assemblies differentially expressed 8-fold at time point comparisons 0 vs 24, 0 vs 36, 0 vs 48, and 0 vs 72 h in immature zygotic embryo explants of maize in inbred line A188 at 0, 24, 36, 48, and 72 h after placement on tissue culture initiation medium.
(XLSX)

Table S5 **Gene ontology describing genes differentially expressed compared to control.** Gene ontology and enrichment summaries for genes that displayed an 8-fold expression change or greater at time point comparisons 0 vs 24, 0 vs 36, 0 vs 48, and 0 vs 72 h in immature zygotic embryo explants of maize in inbred line A188 at 0, 24, 36, 48, and 72 h after placement on tissue culture initiation medium.
(XLSX)

Table S6 **K-means clustering of genes expressed in early somatic embryogenesis.** K-means clustering results of genes expressed in immature zygotic embryo explants of maize in inbred line A188 at 0, 24, 36, 48, and 72 h after placement on tissue culture initiation medium was performed allowing 6 clusters and gene annotations based on MapMan 5b filtered gene set gene names and annotations for the joint loci representative transcript assemblies (RTA).
(XLSX)

Table S7 **Gene onotology describing genes grouped by k-means clustering.** Gene ontology enrichment analysis for genes in one of six clusters as determined by k-means analysis of transcripts detected in immature zygotic embryo explants of maize in inbred line A188 at 0, 24, 36, 48, and 72 h after placement on tissue culture initiation medium.
(XLSX)

Table S8 **Results of coexpression analysis between somatic embryogenesis-related genes.** Coexpression analysis was done with transcripts detected in early somatic embryogenesis of the maize inbred line A188 at 0, 24, 36, 48, and 72 h after placement of explant tissues on tissue culture medium where select maize genes were determined as subjects such as maize genes with high sequence similarity to BABY BOOM1 (BBM1), LEAFY COTYLEDON2 (LEC2), and CLAVATA1 (CLV1), and maize homologs for germin-like protein 2-1 (ZmGLP2-1), glutathione S-transferase 18, 19, and 22 (ZmGST 18, ZmGST 19, ZmGST22), LEAFY COTYLEDON1 (ZmLEC1), PINFORMED1 (ZmPIN1a), SOMATIC EMBRYOGENESIS-LIKE KINASE1 and 3 (ZmSERK1 and ZmSERK3), WUSCHEL1 (ZmWUS1), and Wuschel-related homeobox domain 5 (ZmWOX5B) were used to determined corresponding maize genes as targets with a correlation co-efficient greater than and equal to 0.75.
(XLSX)
