# Supplementary figures and images for: Whole Transcriptome Profiling of Maize during Early Somatic Embryogenesis Reveals Altered Expression of Stress Factors and Embryogenesis-Related Genes
Source: PLoS One. 2014 Oct 30;9(10):e111407. doi: 10.1371/journal.pone.0111407 (PMC4214754; doi:10.1371/journal.pone.0111407)

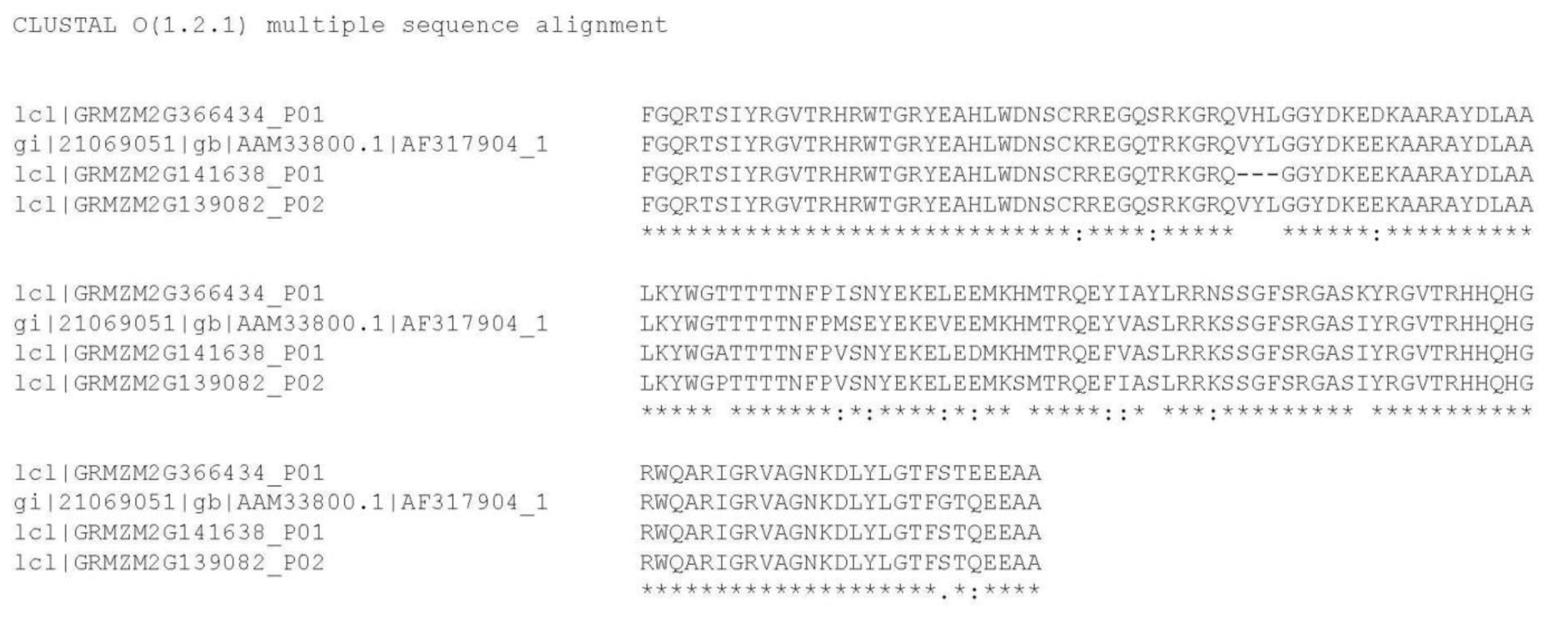

Supplement: Figure S1 — Sequence alignment to determine sequence similarity of BABY BOOM in maize. Multiple sequence alignment of the conserved 147 amino acid sequence of BABY BOOM1 (gene accession AF317904) and three maize annotated proteins with high sequence similarity: GRMZM2G366434_P01, GRMZM2G141638_P01, and GRMZM2G139082_P02. (TIF) [file pone.0111407.s001.tif]

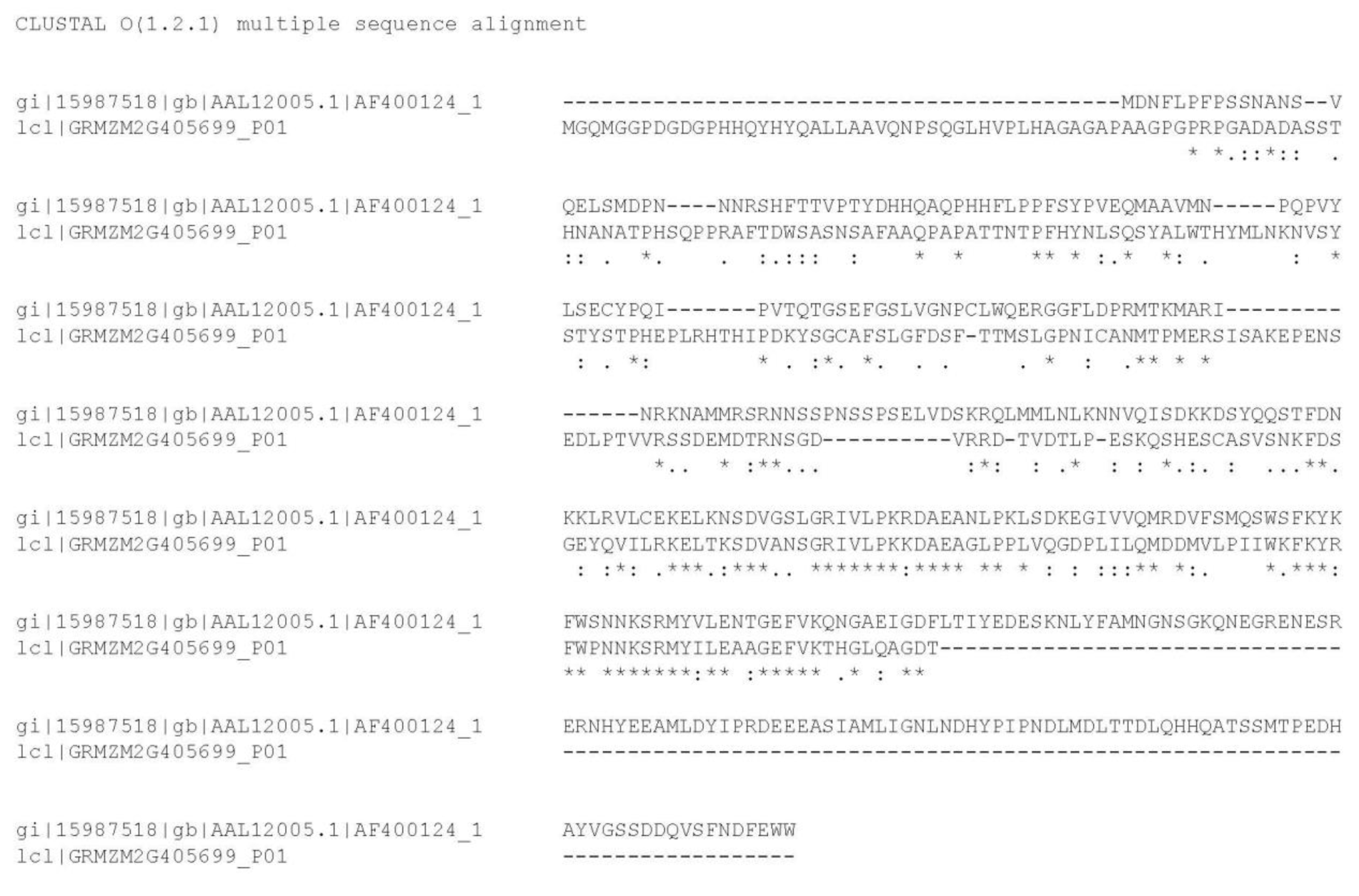

Supplement: Figure S2 — Sequence alignment to determine sequence similarity of LEAFY COTYELODN2 in maize. Sequence alignment between LEAFY COTYELODN2 in Arabidopsis (gene accession AF400124) and maize protein GRMZM2G405699_P01. (TIF) [file pone.0111407.s002.tif]

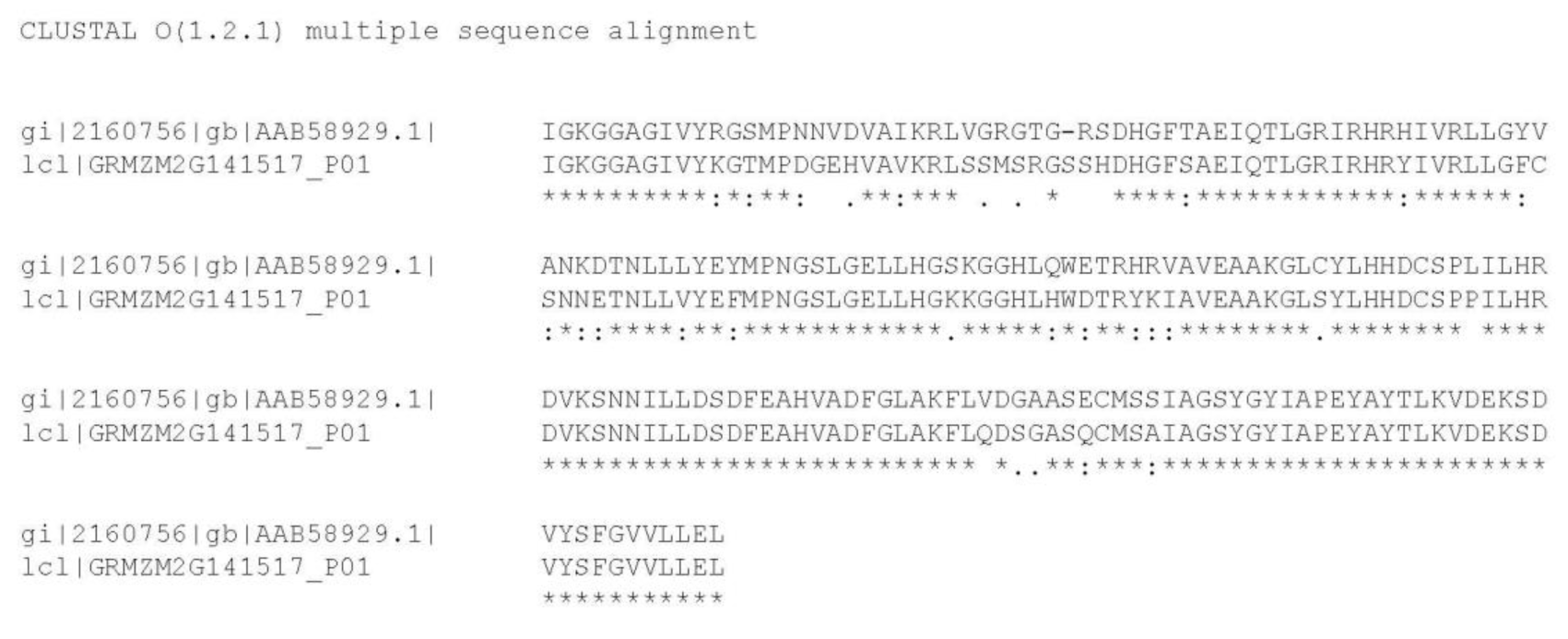

Supplement: Figure S3 — Sequence alignment to determine sequence similarity of CLAVATA in maize. Sequence alignment of the 191 amino acid translated sequence representing the catalytic domain of protein kinases superfamily of CLAVATA (CLV1) in Arabidopsis (gene accession U96879) and the maize protein GRMZM2G141517_P01. (TIF) [file pone.0111407.s003.tif]

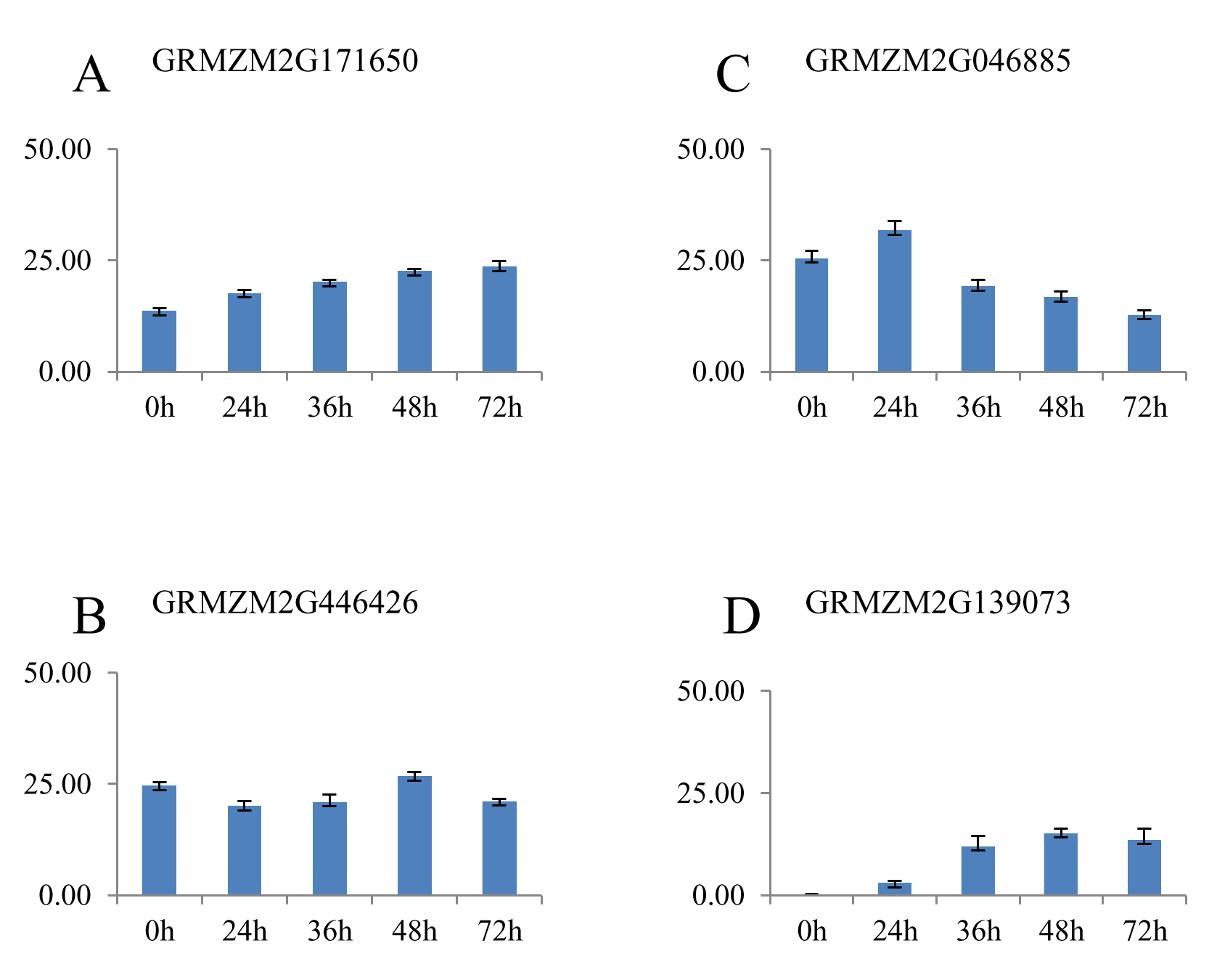

Supplement: Figure S4 — Gene expression trends in early somatic embryogenesis of maize genes that are similar to AGL15. Fragments per kilobase of exon model per million fragments mapped (FPKM) of maize genes with high sequence similarity to Arabidopsis gene AGL15 (gene accession U22528) associated with embryogenic callus induction expressed in immature zygotic embryos of maize inbred line A188 at 0, 24, 36, 48, and 72 h after placement on culture initiation medium. Genes shown include (A) ZmMADS69 (GRMZM2G171650), (B) ZmMADS52 (GRMZM2G446426), (C) ZmMADS73 (GRMZM2G046885), and (D) SILKY1 (GRMZM2G139073). (n = 4 for 0, 24, 36, and 48 h include technical and biological replicates; n = 2 for 72 h include only technical replicates). (TIF) [file pone.0111407.s004.tif]
